# Predictors of mortality in renal transplant recipients with severe sepsis and septic shock

**DOI:** 10.1186/cc12936

**Published:** 2013-11-05

**Authors:** Mônica Andrade de Carvalho, José Osmar Medina Pestana, Flávio Geraldo Rezende Freitas, Flávia Ribeiro Machado, Hélio Tedesco Silva Júnior

**Affiliations:** 1 Nephrology Department, Federal University of São Paulo, Brazil; 2 Anesthesiology, Pain and Intensive Care Department, Federal University of São Paulo, Brazil

## Background

Renal transplantation is the treatment of choice for end-stage renal disease as it is cost-effective, and improves survival and quality of life as compared with maintenance dialysis [1,2]. However, the need for immunosuppression increases the hazard of septic complications [3]. Sepsis is one of the leading causes of death among renal transplant recipients and little is known about its characteristics in this population [4,5]. The aim of this study was to evaluate the factors associated with mortality in renal transplant patients admitted to the ICU with severe sepsis and septic shock.

## Materials and methods

We conducted a single-institution retrospective observational cohort study in consecutive renal transplant patients admitted to the ICU with severe sepsis or septic shock in a public high-volume kidney transplant center from 1 June 2010 and 31 December 2011. We registered demographic data, transplant characteristics and sepsis management to identify predictive factors of ICU, hospital and 1-year mortality.

## Results

A total of 190 patients were enrolled. The mean age was 51 ± 13 years, 115 (60.5%) were male, 122 (64.2%) were deceased donors, median APACHE was 20 (16 to 23) and median admission SOFA was 5 (4 to 8). The most common source of infection was respiratory (59.5%) followed by urinary tract (16.8%). Tachypnea, tachycardia, fever, hypothermia, leukocytosis and leukopenia were present in 74.7%, 67.9%, 24.2%, 6.3%, 26.3% and 16.3% of the patients. The most prevalent dysfunction was respiratory (68.4%) followed by cardiovascular (41.1%) and renal (40.5%). The median time between transplantation and the septic event was 2.1 (0.6 to 7.8) years. The duration of organ dysfunction before the diagnosis of sepsis was 2.5 (1.1 to 5.2) hours. The median length of ICU and hospital stay was 6 (3 to 13) and 20 (12 to 35) days, respectively. Hospital and 1-year mortalities were 38.4% and 42.6%, respectively. In the multivariate analysis, male gender, the variation in the SOFA score after the first 24 hours, the need for mechanical ventilation, the presence of hematologic dysfunction, being admitted from the wards and AKI stage 3 were predictors of hospital mortality.

## Conclusions

In the present study, independent factors associated with mortality were related to features of sepsis severity and not to factors associated with transplantation. Another interesting finding was the low frequency of signs of systemic inflammatory response.
